# Supplementary figures and images for: Genome-wide identification of C2H2 zinc-finger genes and their expression patterns under heat stress in tomato (Solanum lycopersicum L.)
Source: PeerJ. 2019 Nov 25;7:e7929. doi: 10.7717/peerj.7929 (PMC6882421; doi:10.7717/peerj.7929)

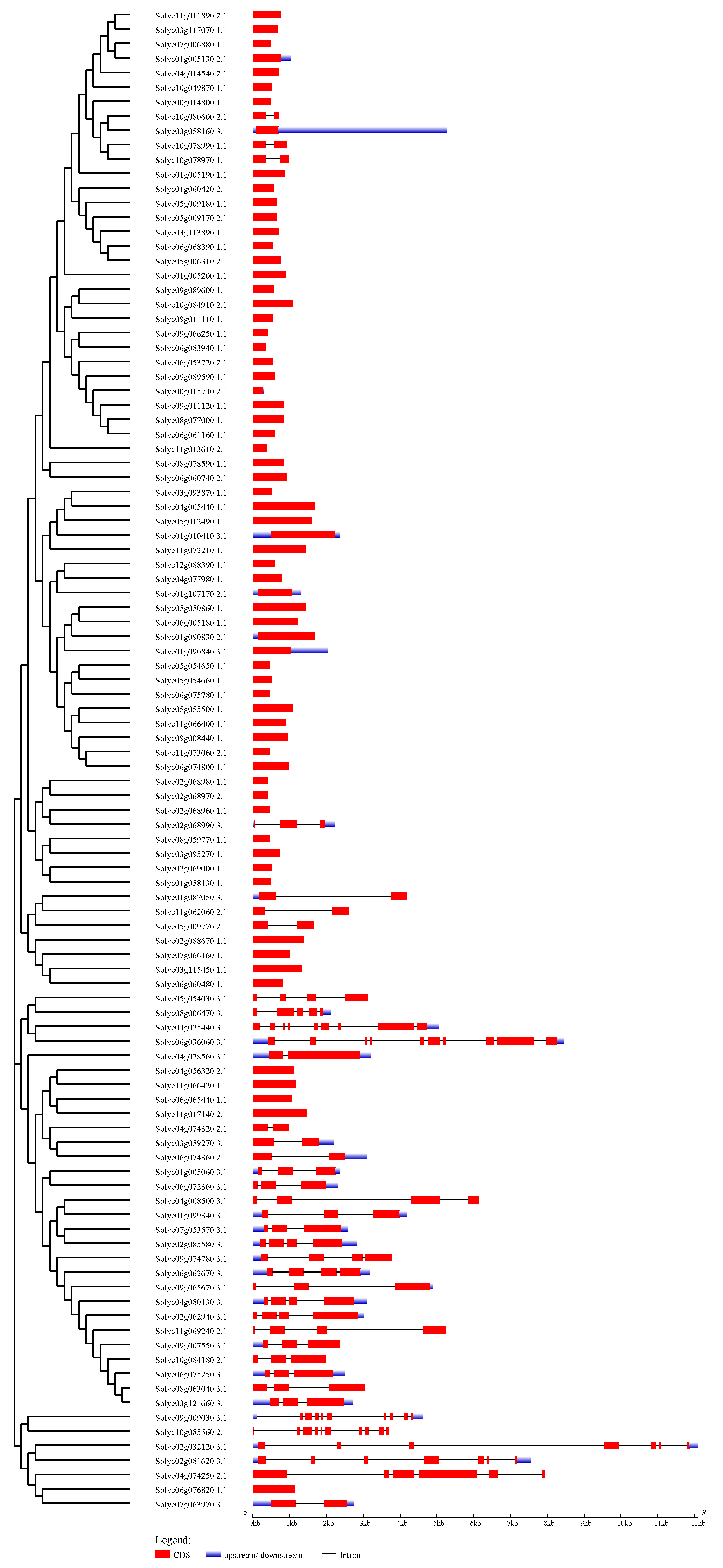

Supplement: Figure S1A — The Maximum Likelihood tree was created using the ProtML program under the JTT model (bootstrap value set at 1000). The coding sequences (CDSs), untranslated regions (UTRs), and introns are depicted by filled red boxes, blue boxes, and single black lines, respectively. The scale bar indicates the length of the corresponding genes (kb). [file peerj-07-7929-s002.jpg]

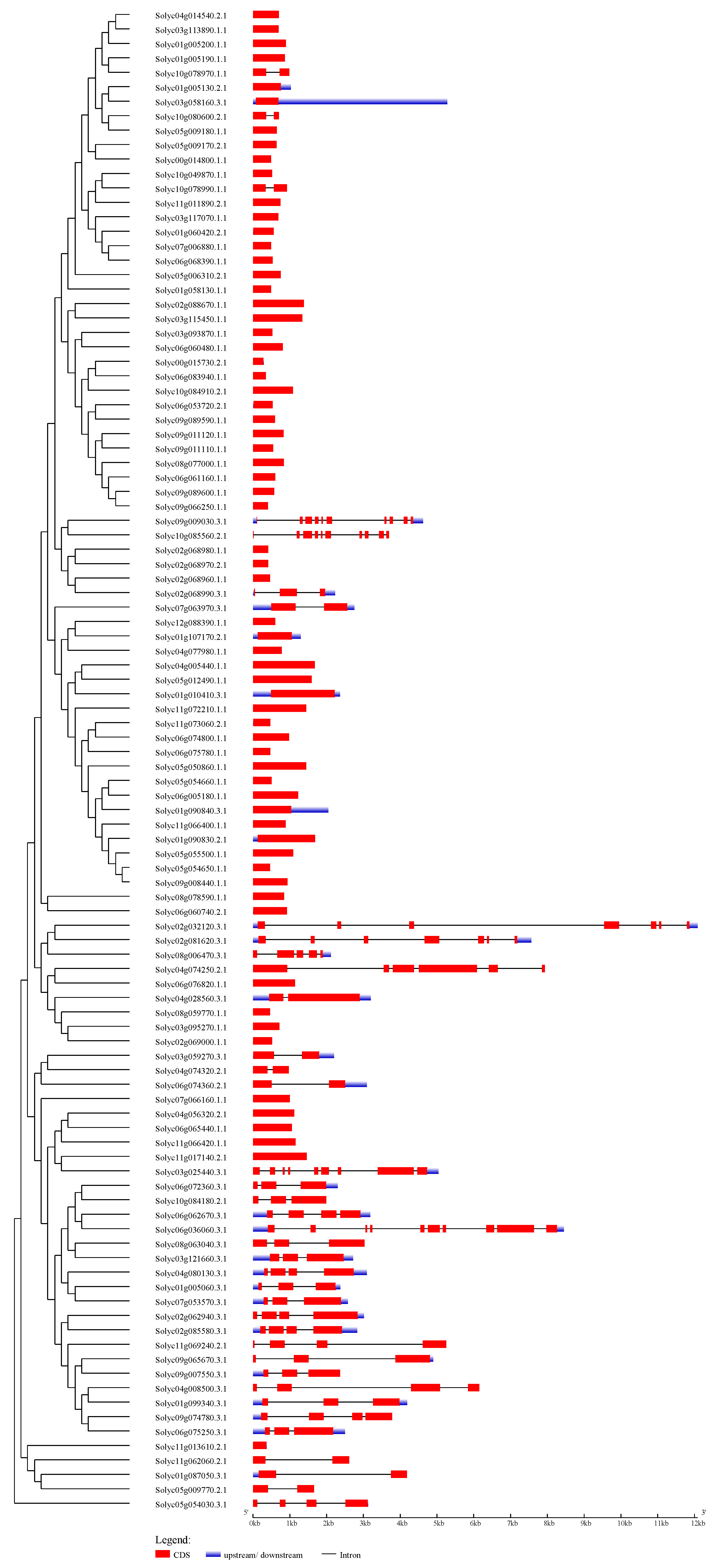

Supplement: Figure S1B — The UPGMA tree was created using the MEGA6.0 program (bootstrap value set at 1000). The coding sequences (CDSs), untranslated regions (UTRs), and introns are depicted by filled red boxes, blue boxes, and single black lines, respectively. The scale bar indicates the length of the corresponding genes (kb). [file peerj-07-7929-s003.jpg]

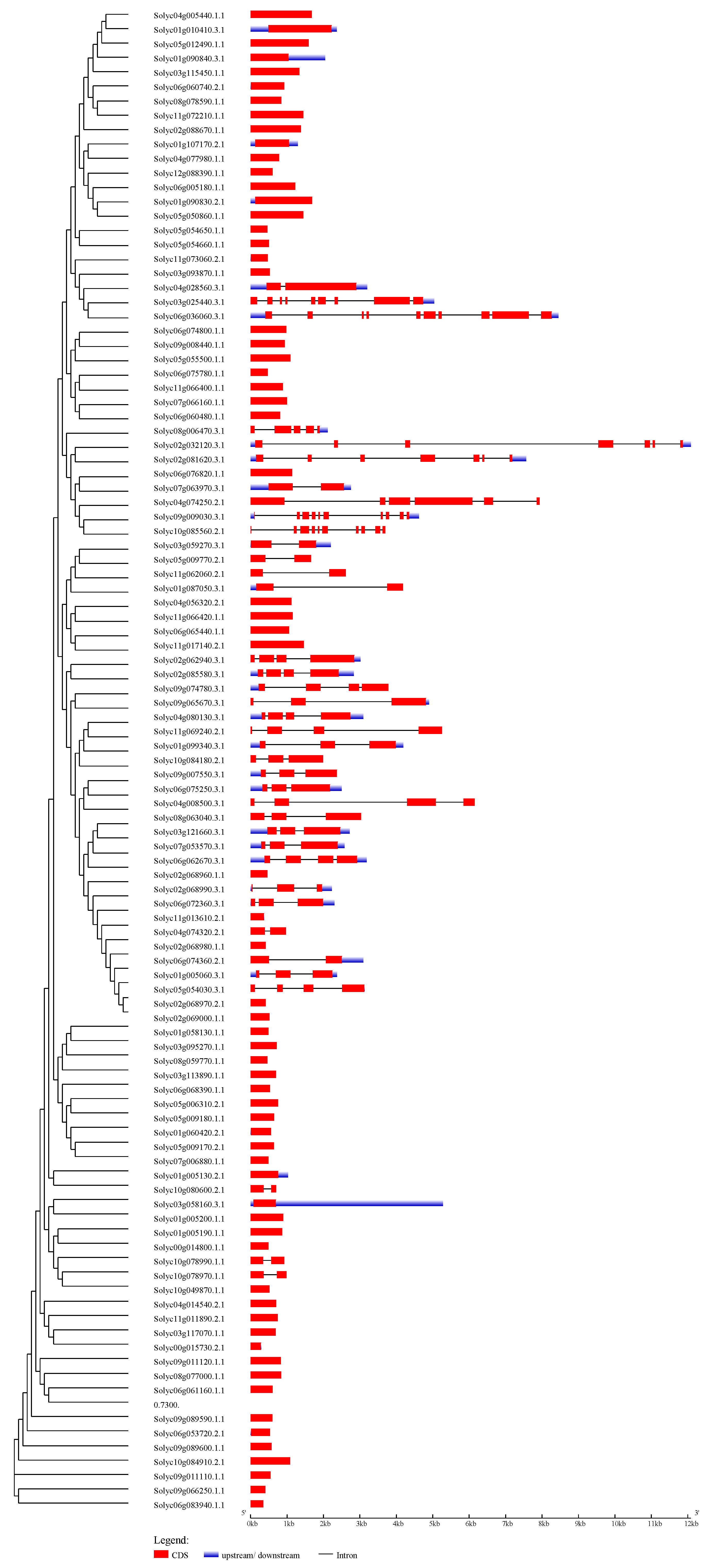

Supplement: Figure S1C — The UPGMA tree was created using the MEGA6.0 program (bootstrap value set at 1000). The coding sequences (CDSs), untranslated regions (UTRs), and introns are depicted by filled red boxes, blue boxes, and single black lines, respectively. The scale bar indicates the length of the corresponding genes (kb). [file peerj-07-7929-s004.jpg]

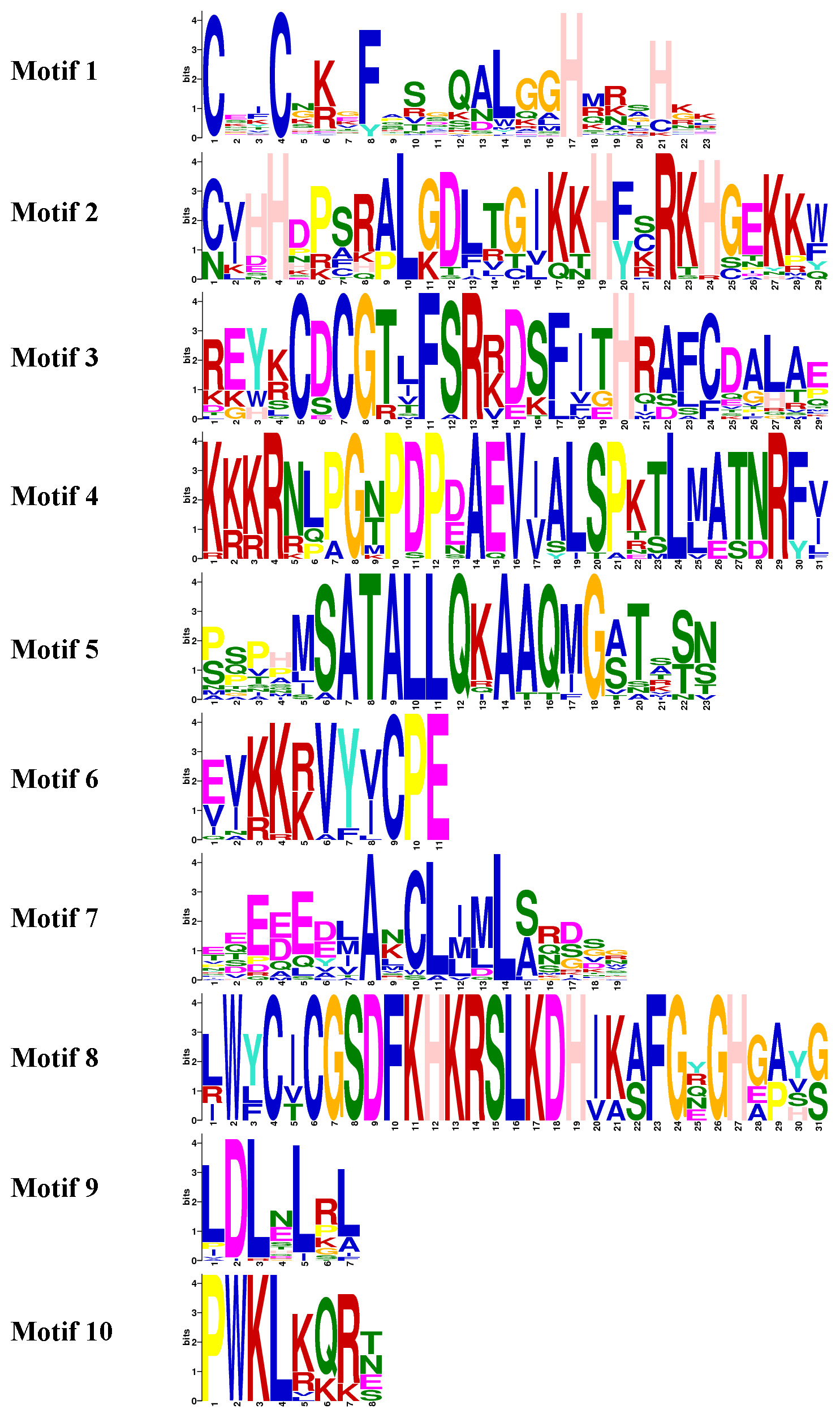

Supplement: Figure S2 — The sequence information for each motif is represented by motif logos. [file peerj-07-7929-s005.jpg]

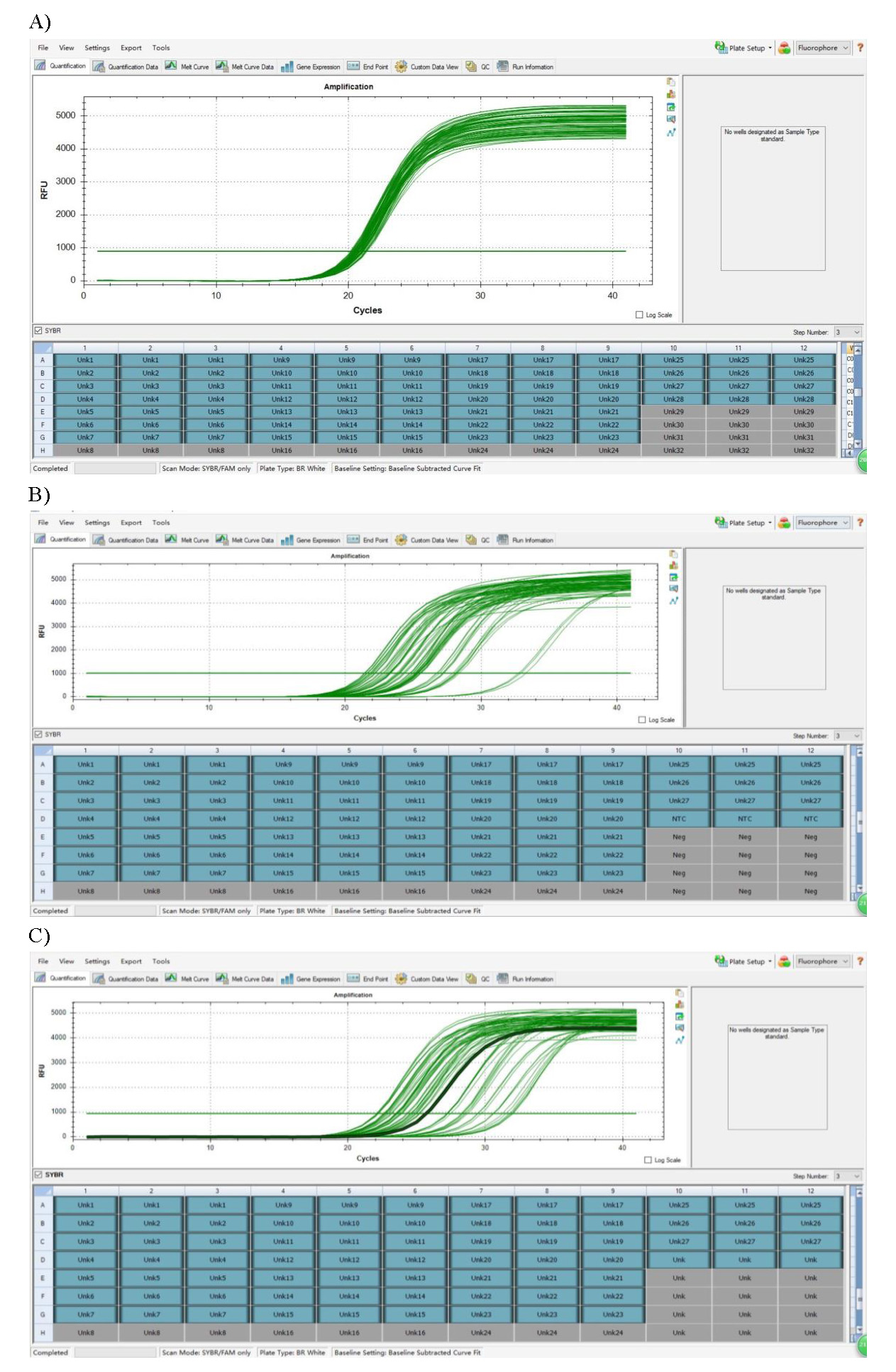

Supplement: Figure S3 — (A) SlEF1-α gene (Solyc06g005060); (B) SlACT (Solyc03g078400); (C) SlUBI3 (Solyc01g056940). [file peerj-07-7929-s006.jpg]

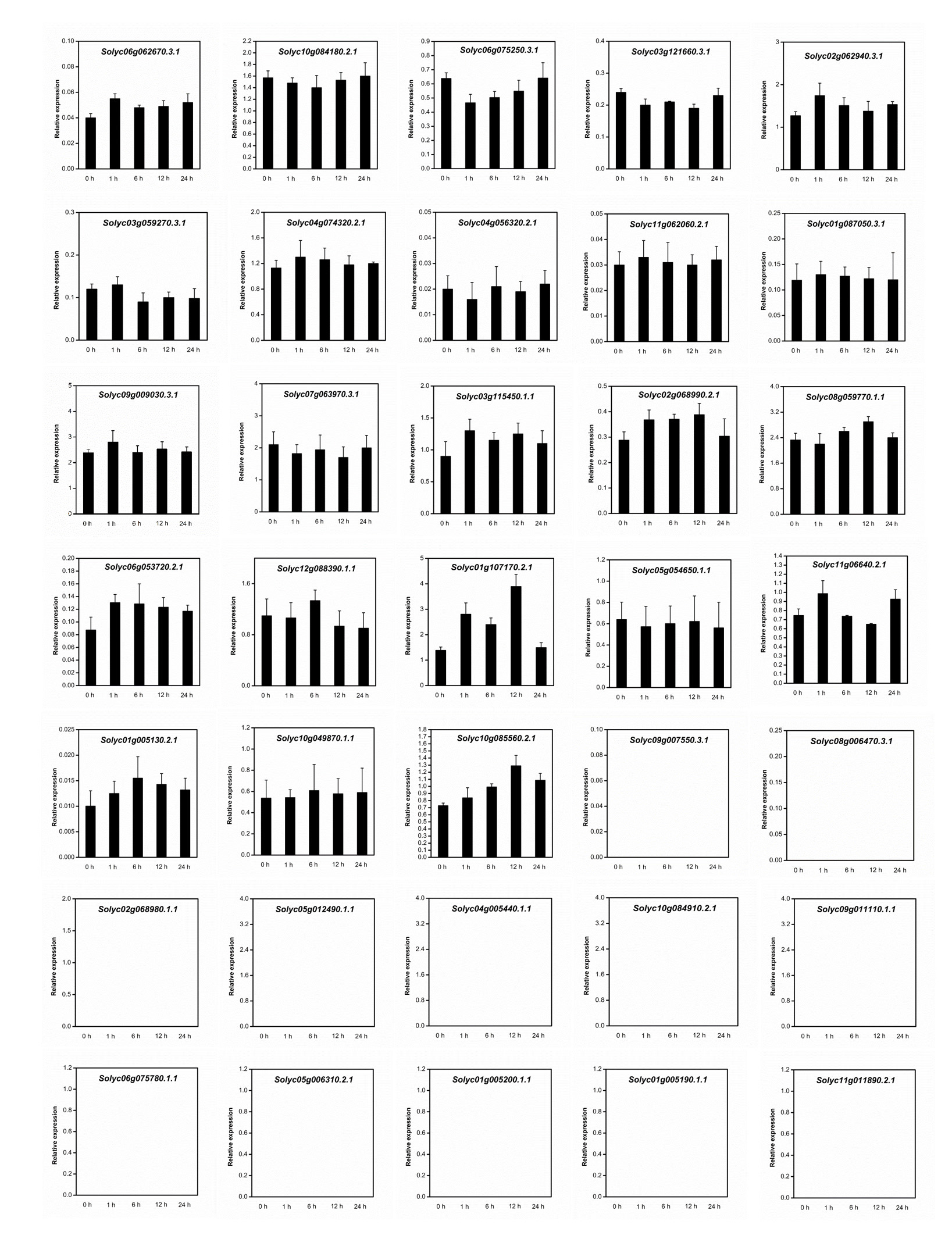

Supplement: Figure S4 — The relative expression levels were normalized to the reference gene (SlEF1-α; Solyc06g005060). Error bars represent the standard deviations from three biological replicates. [file peerj-07-7929-s007.jpg]

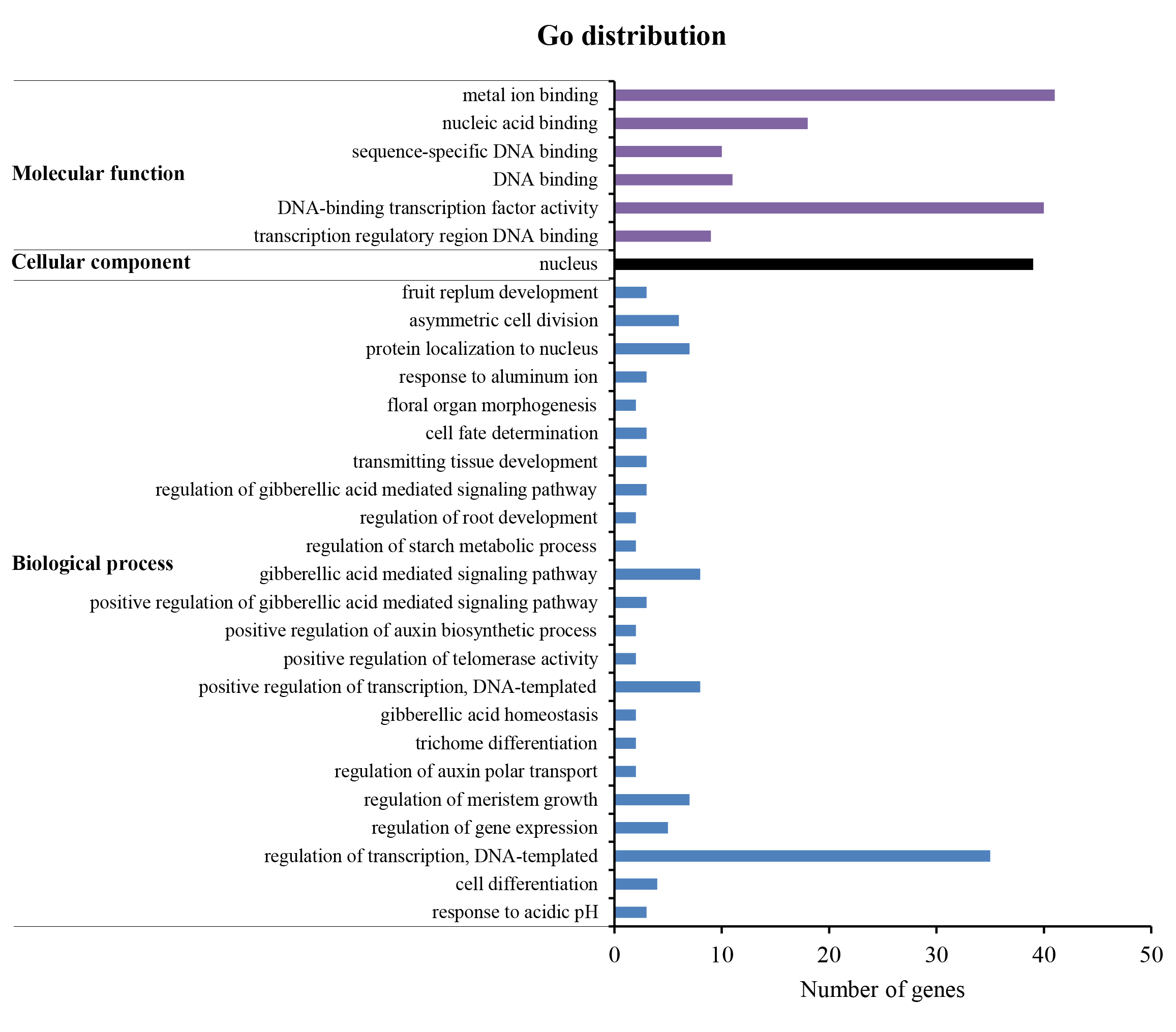

Supplement: Figure S5 — The annotation results were classified into three GO categories on the basis of GO second level terms. [file peerj-07-7929-s008.jpg]
